# The complete chloroplast genome and phylogenetic analysis of *Ocimum kilimandscharicum* Gurke (Camphor Basil) from India

**DOI:** 10.1080/23802359.2021.1945505

**Published:** 2021-07-01

**Authors:** Samuel Yesuthason Renald, Raju Balaji, Tanuja, Madasamy Parani

**Affiliations:** Center for DNA Barcoding, Department of Genetic Engineering, SRM Institute of Science and Technology, Kattankulathur, India

**Keywords:** Complete chloroplast genome, *Ocimum kilimandscharicum*, Camphor Basil, Lamiaceae, Karpoora Tulsi

## Abstract

*Ocimum kilimandscharicum* Gurke commonly known as Camphor Basil, is a medicinal plant species that belongs to the Lamiaceae family. Here, the sequencing and characterization of complete chloroplast genome sequence of *O. kilimandscharicum* is reported for the first time using Illumina paired-end sequencing data. The size of the chloroplast (cp) genome is 151,741 bp in length, with a large single-copy (LSC) region of 82,882 bp and a small single-copy (SSC) region of 17,587 bp, separated by a pair of 25,636 bp inverted repeat (IR) regions. There are 135 predicted genes, including 90 protein-coding genes, 37 transfer RNA (tRNA) genes, and eight ribosomal RNA (rRNA) genes in the genome, and the overall GC content of the genome is 37.9%. The phylogenetic analysis based on the chloroplast genome data indicated that *O. kilimandscharicum* is closer to *O. tenuiflorum* and clustered to other *Ocimum* species in Lamiaceae.

*Ocimum kilimandscharicum* Gurke commonly known as Camphor Basil or Karpoora Tulsi in Tamil, belongs to Lamiaceae family. It is a shrub native to East Africa, which was brought to India and used to treat constipation, abdominal pain, cough, measles, and diarrhea (Gill et al. 2012). The leaves are rich in essential oil (0.004 − 0.7%), and camphor is the major component used in perfumery, flavor, and pharmaceutical industries (Lima et al. 2014). This plant is used in traditional medicine for its various pharmacological activities, including antioxidant, antimicrobial, wound healing, anti-malarial, antifungal, anti-nociceptive, anti-diarrhoeal, and insecticidal properties (Sarin et al. 2013). To provide genomic resources for investigating the evolution of *O. kilimandscharicum*, we sequenced the complete chloroplast genome sequence of this species and studied its phylogenetic relationships within the Lamiaceae family.

Fresh leaves of *O. kilimandscharicum* were collected from the Foundation for Revitalization of Local Health Traditions (FRLHT), Yelahanka, Bengaluru, Karnataka, India (GPS: 13°07'24.5"N 77°32'52.3"E). The voucher specimen was deposited at the SRM Institute of Science and Technology Herbarium (http://www.srmist.edu/, Dr. P. Senthilkumar, senthilp3@srmist.edu.in) under the voucher number SRMH000145. The total genomic DNA was extracted from fresh leaves as described before (Vassou et al. 2015) and stored at −80˚C until use. A whole-genome DNA sequencing library was constructed using the Nextera XT Library Prep Kit. The DNA library was sequenced on NextSeq 500 platform (Illumina Inc., San Diego, CA), and we obtained 2.5 Gb of paired-end sequencing data. The chloroplast genome of *O. kilimandscharicum* was assembled with 984x coverage using NovoPlasty v.4.3.1 (k-mer 33) (Dierckxsens et al. 2017) with *O. gratissimum* L. (Balaji et al. 2021) as a reference seed sequence. The assembled *O. kilimandscharicum* chloroplast genome was annotated by GeSeq (Tillich et al. 2017) using the chloroplast genomes of *O. tenuiflorum* (NC_043873.1) and *O. basilicum* (NC_035143.1) as reference sequences. The predicted transfer RNAs (tRNAs) were confirmed by tRNAscan-SE 2.0 (Lowe and Chan 2016).

The complete cp genome of *O. kilimandscharicum* (GenBank Accession No. MW829603) exhibits a typical quadripartite circular molecule with 151,741bp genome size. It is composed of a large single-copy (LSC) region of 82,882 bp, a small single-copy region (SSC) of 17,587 bp, and a pair of inverted repeat (IR) regions of 25,636 bp. The GC content of the complete chloroplast genome was 37.9%, while the LSC, SSC, and IR regions are 36.0%, 31.8%, and 43.2%, respectively. The cp genome encodes an identical set of 135 predicted functional genes, including 90 protein-coding genes, 37 transfer RNA (tRNA) genes, and eight ribosomal RNA (rRNA) genes.

A maximum-likelihood tree of molecular distance analysis was performed using 1000 bootstrap replicates with (GTR + G) model in MEGA version X (Kumar et al. 2018) from the alignments created using the MAFFT program (Katoh and Standley 2013). *Nicotiana tabacum* (Solanaceae) and *Arabidopsis thaliana* (Brassicaceae) were designated as outgroup taxa, and 16 published complete chloroplast genome sequences from the Lamiaceae family were included as in-group taxa. The phylogenetic analysis of *O. kilimandscharicum* revealed that it is closely related to *O. tenuiflorum*, *O. gratissimum,* and *O. basilicum* (Figure 1). This study enriches the genetic resources of *O. kilimandscharicum* and contributes to its species delimitation, phylogenetic analysis, DNA barcoding, and evolutionary relation of genus *Ocimum* and Lamiaceae in the future.

**Figure 1.**
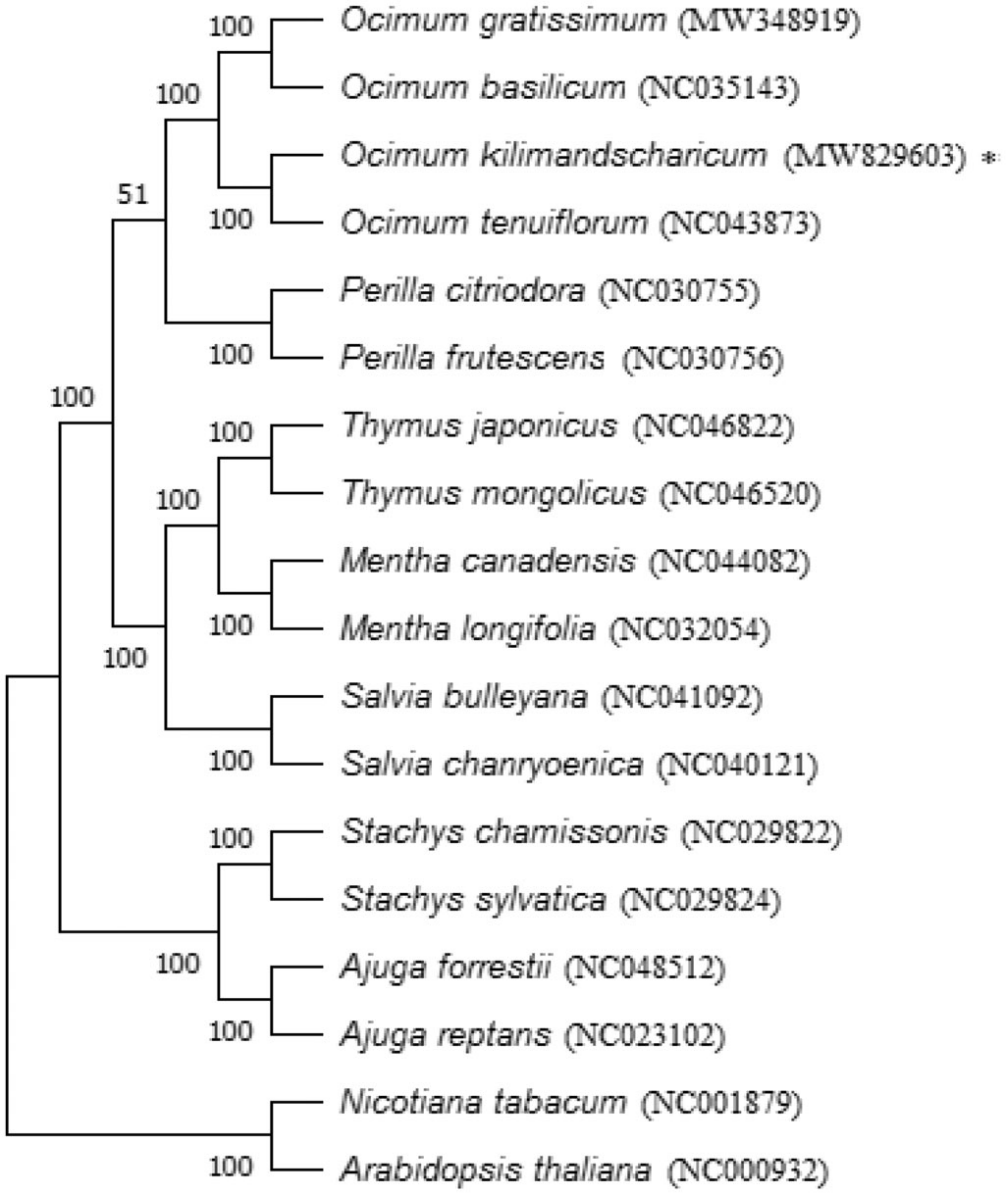
Phylogenetic tree reconstructed by maximum likelihood (ML) analysis based on chloroplast genome sequences, including *O. kilimandscharicum* (MW829603)* sequenced in this study. The bootstrap support values >50% from 1,000 replicates are indicated in the nodes.

## Data Availability

The data that support the findings of this study are openly available in NCBI at https://www.ncbi.nlm.nih.gov/nuccore/MW829603.1/, under GenBank accession number MW829603. The NGS sequencing data are available from the BioProject, SRA, and Bio-Sample ID under the accession numbers PRJNA717657, SRR14076648, and SAMN18499778 respectively.
